# Micro-structures, nanomechanical properties and flight performance of three beetles with different folding ratios

**DOI:** 10.3762/bjnano.13.75

**Published:** 2022-08-26

**Authors:** Jiyu Sun, Pengpeng Li, Yongwei Yan, Fa Song, Nuo Xu, Zhijun Zhang

**Affiliations:** 1 Key Laboratory of Bionic Engineering (Ministry of Education, China), Jilin University, Changchun, 130022, P.R. China; 2 Key Laboratory of CNC Equipment Reliability (Ministry of Education) and School of Mechanical and Aerospace Engineering, Jilin University, Changchun, 130022, P.R. China

**Keywords:** beetle hind wings, flight performance, folding ratio, nanoindentation, wind tunnel

## Abstract

When beetles are not in flight, their hind wings are folded and hidden under the elytra to reduce their size. This provided inspiration for the design of flapping-wing micro aerial vehicles (FWMAVs). In this paper, microstructures and nanomechanical properties of three beetle species with different wing folding ratios living in different environments were investigated. Factors affecting their flight performance, that is, wind speed, folding ratio, aspect ratio, and flapping frequency, were examined using a wind tunnel. It was found that the wing folding ratio correlated with the lift force of the beetles. Wind speed, folding ratio, aspect ratio, and flapping frequency had a combined effect on the flight performance of the beetles. The results will be helpful to design new deployable FWMAVs.

## Introduction

Regarding the benefits of scientific research, rescue, surveying, mapping, and many other aspects in the development of micro aerial vehicles (MAVs), miniaturization of aircraft has become a popular research topic [1]. Owing to their small size, light weight, flexibility and concealment, MAVs have completed a series of tasks that are difficult or impossible for larger aircraft [2]. Due to unsteady aerodynamic effects, flapping wings may be a more energy-efficient flight mode than modes achieved with traditional fixed and rotor wings [3–6]. The lift-to-power metric of the revolving wing declines rapidly with decreasing Reynolds numbers, resulting in a hovering performance that is at least a factor of two lower than the flapping wing at Reynolds numbers less than about 100 [7]. Flapping-wing micro-aerial vehicles (FWMAVs) are increasingly favored by scientists worldwide for their excellent flight performance due to unsteady aerodynamic mechanisms in the low Reynolds number range [8–10]. In the application of MAVs, flapping wings are becoming more common [11]. Deployable FWMAVs make MAVs smaller in size, lighter, and harder to detect [12–13].

Insects can hover, fly in any direction, turn quickly in the air, and resist interference caused by the external environment, showing strong agility, maneuverability, and stability. This has raised great interest to study the mechanism of the high lift generated by insects in flight and to imitate the flight of insects [14–15]. Insect wings play a major role here. Hence, examining their flight parameters is crucially important to design biomimetic FMAVs [16–17]. It is increasingly clear that most insects obtain useful force with the help of aerodynamic mechanisms that require torsion, often with transverse bending, and other deformations including alteration of the effective area [18]. The butterfly can increase the aspect ratio by spreading the forewings to generate maximum lift and increase the lift-to-drag ratio during flapping flight. Thus, it significantly enhances the maneuverability through rapidly changing the flight speed by changing its flapping frequency and amplitude [19]. In studies regarding the flapping frequencies and angles of wings and the lift characteristics of *Anax goliathus*, *Trypoxylus dichotomus*, and *Oncotympane maculaticollis*, it was found that their flapping lifts were different because of the different sizes and shapes of the wings [20]. Additionally, the elasticity of insect wings also has an impact on the aerodynamic characteristics. By studying the flexible deformations and aerodynamic characteristics of cicada wings during flapping, it was found that their flexibility can increase their mean lift coefficient [21]. In rhinoceros beetles, the elytra is also involved in aerodynamics during takeoff, producing an interaction force between the elytra and the hind wings [22].

To avoid damage or hinder the movement on the ground, the hind wings of beetles are folded to reduce their size and to hide under the elytra [23–24]. The foldable wings of beetles have attracted the interest of aerospace engineering scientists as well as entomologists [25]. Knowledge about the folding mechanism of beetle hind wings can be used to design deployable FWMAVs [16,26]. Rapidly deployable wings based on the design of origami mechanisms [27] and foldable artificial wings imitating *Allomyrina dichotoma* have opened up new prospects for foldable structures in MAVs [28]. The folding pattern and geometric and kinematic parameters of beetle hind wings have a critical effect on the flight performance and will be useful regarding the design of biomimetic MAVs.

Wind tunnels are an effective tool to investigate flapping-wing flight and aerodynamic characteristics [29]. The wing tip trajectories of the hind wings of a beetle species (*Protaetia brevitarsis*) were captured during wind tunnel tests and, based on the results, bioinspired wings and a linkage-mechanism flapping system were designed [26]. The flow behavior of live rhinoceros beetle flapping hind wings was studied by using a smoke wind tunnel and a high-speed camera [30]. Combining wind tunnels and high-speed cameras, the relationships between wing shapes, flapping flight lift, and aerodynamic efficiency of dragonflies [31] and fruit flies [32] were examined. In addition, the influence of frequency and wind speed on the aerodynamic characteristics (such as the lift, drag, and vortex) of MAV prototypes with different wing types were also tested in wind tunnels [33–36].

Various beetle species have different hind wing folding methods, and their folding ratios (i.e., the ratio between folding length of the hind wings and the length of the hind wings) are different. In this paper, three beetle species from different living environments were selected to explore the influence of different folding ratios on their flight performance through wind tunnel tests. The aspect ratios and flapping frequencies and the influence of the flow velocity on their lift-to-drag ratio are discussed in the following, which will provide some reference for the design of biomimetic deployable FWMAVs.

## Materials and Methods

### Beetles

Three beetle species with different folding ratios, *Protaetia brevitarsis*, *Anoplophora chinensis*, and *Trypoxylus dichotomus*, were selected for the experiments. The three adult *P. brevitarsis* used in the experiment were captured in Nanchang, Jiangxi Province, China, and the three adult *A. chinensis* and the three adult *T. dichotomus* were captured in Nanyang, Henan Province, China. The parameters of hind wings of the three beetle species are shown in Table 1. The body length of *P. brevitarsis* was 21.41 ± 1.58 mm, the body width was 13.09 ± 0.55 mm, and the body weight was 0.72 ± 0.13 g. The body length of *A. chinensis* was 27.65 ± 2.54 mm, the body width was 10.28 ± 1.07 mm, and the body weight was 1.01 ± 0.35 g. The body length of *T. dichotomus* was 43.96 ± 2.42 mm, the body width was 23.74 ± 1.53 mm, and the body weight was 5.27 ± 0.16 g. All insects were acclimated under standard laboratory conditions (ventilation room, 25 ± 1 °C, 60% ± 5% humidity, 12 h light/dark cycle) and had free access to standard water and food. All procedures were conducted in accordance with the “Guiding Principles in the Care and Use of Animals” (China) and were approved by the ethics committee of experimental animal welfare of Jilin University.

**Table 1:** Beetle hind wing parameters.

Beetle	*P. brevitarsis*	*A. chinensis*	*T. dichotomus*

body weight (g)	0.72 ± 0.13	1.01 ± 0.35	5.27 ± 0.16
hind wing area (mm^2^)	181.341 ± 15.35	238.02 ± 56.63	801.72 ± 111.75
hind wing extented length (mm)	26.47 ± 1.36	29.34 ± 4.32	53.60 ± 4.28
hind wing folded length (mm)	11.58 ± 0.76	9.98 ± 1.46	22.33 ± 2.15
folding ratio	0.44	0.34	0.42
flapping frequency (Hz)	90	45	43
aspect ratio	3.86	3.64	3.59
wing loading (g/dm^2^)	19.85	21.22	32.87

### High-speed camera

A high-speed camera (Phantom V711, Vision Research Inc., USA) was used to obtain the postures and flapping frequencies of the three beetles during flight. A camera speed of 2000 frames/s (shutter speed: 0.1 ms, resolution: 1280 × 800 pixels) was used to measure the flapping period of the beetle in flight and to determine its flapping frequency.

### Microscopic morphologies of hind wings of beetles

A super depth-of-field microscope (VHX-6000, Keyence, Japan) was used to obtain images of the fully unfolded and folded hind wings of the three beetles. To obtain the macroscopic structures of the hind wings of the three beetles, the hind wings were first removed with a scalpel and rinsed with distilled water and then dried and pasted flat on a slide for observation.

Scanning electron microscopy (SEM) (Model EVO-18, Carl Zeiss Microimaging Inc., Germany) was used to obtain morphological images of cross sections of the hind wings of three beetles at the same locations of different wing veins.

### Nanoindentation properties

The nanomechanical characteristics were tested using a nanoindenter (TriboIndenter, Hysitron Inc., USA). The reduced Young’s modulus, *E*_r_, is calculated as


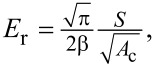


where β is a constant related to the shape of the head (for a Berkovich indenter, the value is 1.034). *A*_c_ is contact the area and a polynomial function of contact depth. *S* is the contact stiffness. A Berkovich tip with a tip radius of approximately 100 nm was used for the tests. In order to study the effect of nanomechanical properties of different veins on the lift of the beetle hind wings, the same location of the same vein of three beetles was selected for testing. Six test positions were selected, namely the anterior of the costal (I), the end of the costal (II), media posterior (III), cubitus anterior (IV), anal posterior (V), and wing membrane (VI) (see below Figure 3). A force of 100 μN was applied to the veins and the loading rate and holding time remained constant at 10 μN/s and 10 s, respectively.

### Wind tunnel

**Figure 1:**
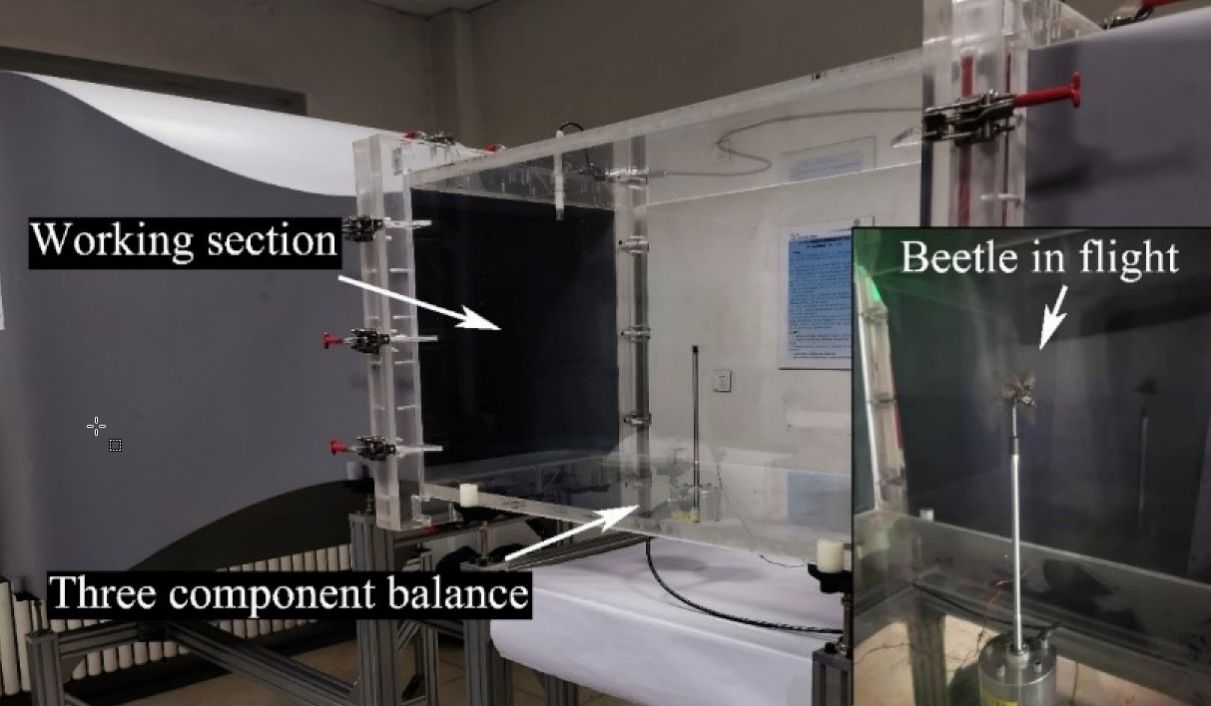
The low-speed straight-flow wind tunnel and the beetle during test.

The wind tunnel test was performed in a low-speed straight-flow wind tunnel at the Key Laboratory of Bionic Engineering, Jilin University, China. The main parameters of the wind tunnel are shown in Table 2. The abdomen of the beetle was attached with AB glue (Epoxy Resin Liquid Adhesive Strong Adhesive, HOU-FC220) to the bracket, which was connected to the force balance (load cell) after adjustment. Flight behavior and body angles of each beetle were ensured to be the same in flight (Figure 1). The selected load cell (LH-SZ-02, Shanghai Liheng, China; 0–20 N ± 0.2 mN) has the advantages of small size, high precision, and fast response, suitable for the flight performance tests of insects and MAVs.

**Table 2:** Main parameters of the wind tunnel.

Test section parameters	Value

working section shape	Rectangle
working section area (mm^2^)	650 × 450
length of working section (mm)	1000
turbulent intensity (%)	<0.3
regulator form of wind speed	Hot-wire sensor
range of wind speed (m/s)	0–10
airflow nonuniformity of working section (%)	<3

Three species of beetles were divided into groups of three. After each beetle was attached to the bracket with AB glue at an angle of attack of 0°, the wind speed was adjusted to 1.0, 1.5, 2.0, 2.5, and 3.0 m/s. Thrust and lift of each beetle at the moment of hover flight at different wind speeds were measured through the force balance. Also, the drag at rest at different wind speeds was measured. After obtaining the lift-to-drag ratio of each beetle at different wind speeds, the average lift-to-drag ratio of each group of beetles was taken as the lift-to-drag ratio of this species of beetles.

## Results and Discussion

### Beetle flying attitude

**Figure 2:**
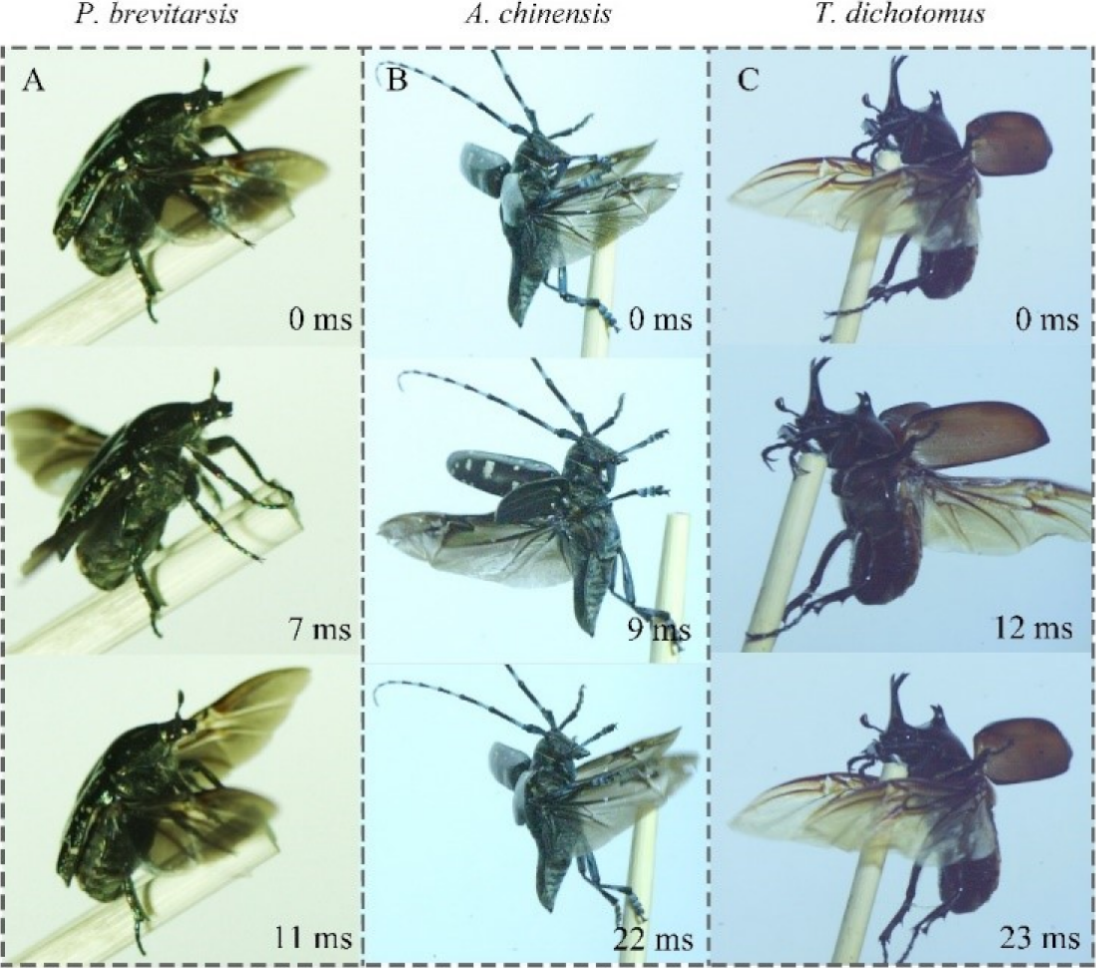
(A), (B), and (C) show one flapping cycle of *P. brevitarsis*, *A. chinensis*, and *T. dichotomus*, respectively.

Figure 2 shows a flapping cycle of the three beetles obtained using a high-speed camera. The flapping frequency of each beetle was calculated by measuring the time required for one flapping cycle. The flapping period of *P. brevitarsis* was 11 ms, and the flapping frequency was 90 Hz. For *A. chinensis*, the values were 22 ms and 45 Hz, respectively, and for *T. dichotomus*, the values were 23 ms and 43 Hz, respectively.

The wingtip path of the upstroke almost overlapped with the wingtip path at the beginning of the downstroke in one flapping cycle. The reason for this is that the beetles needed to rapidly increase the vertical lift at the beginning of the upstroke [37]. The flapping amplitude of the hind wings of *P. brevitarsis* during flight was smaller than that of *A. chinensis* and *T. dichotomus*, which means that the higher flapping frequency was needed [38]. Also, it was found that the flapping amplitude of the elytra of the three beetles will vary with the flapping angle of the hind wings, and the elytra will flap almost synchronous with the hind wings. A similar phenomenon was found in dung beetles (*Heliocopris hamadryas*), whose elytra contributed to produce lift [39]. In addition, the hind wings of beetles twist to a certain extent during flight. The rotational lift is an important factor affecting the aerodynamics of the beetle [30].

### Hind wing unfolding and folding morphology

**Figure 3:**
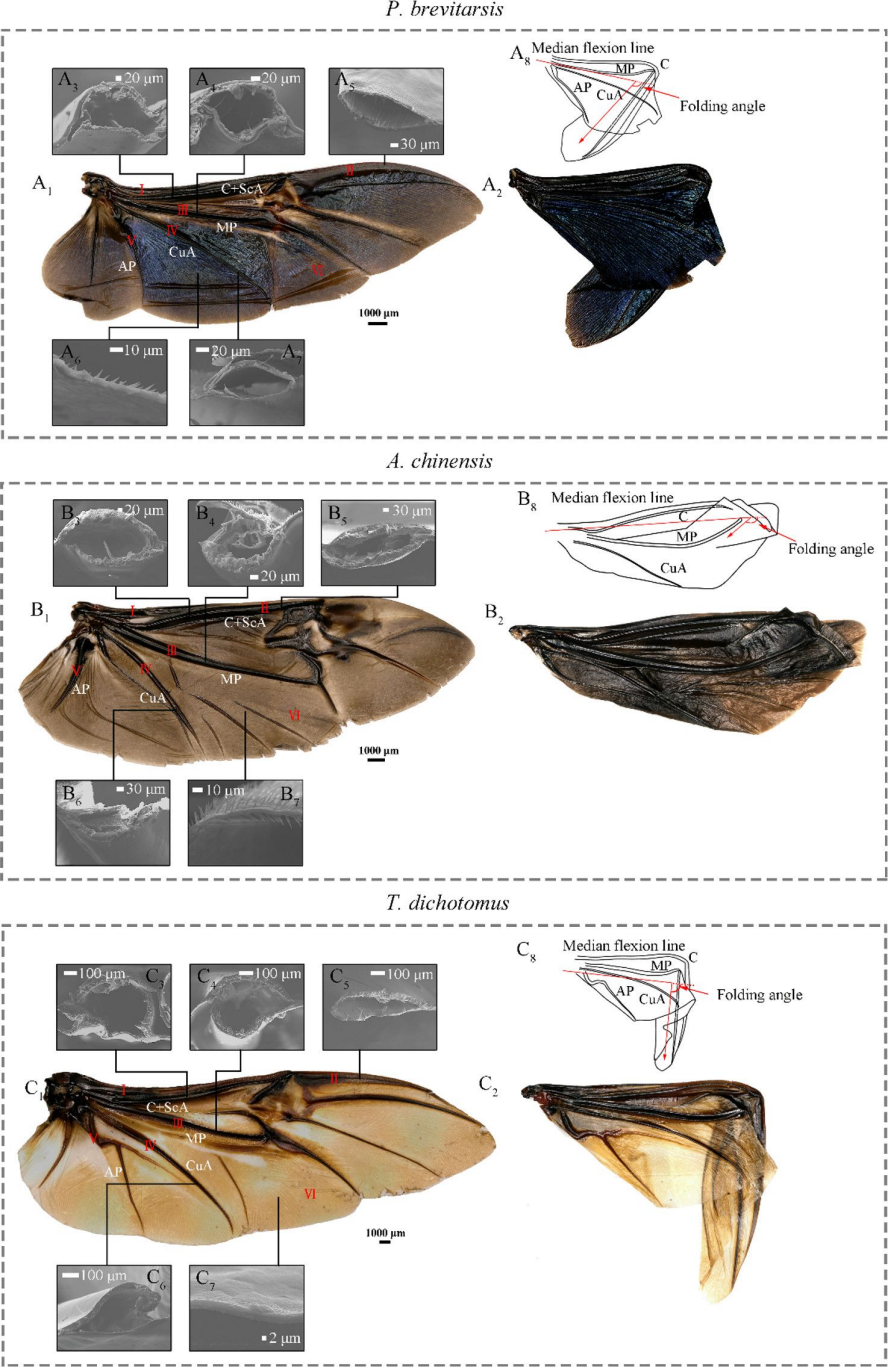
(A), (B), and (C) show hind wings of *P. brevitarsis*, *A. chinensis*, and *T. dichotomus*, respectively. (A_1_) and (A_2_), (B_1_) and (B_2_), and (C_1_) and C_2_ show the extended and folded hind wings of *P. brevitarsis*, *A. chinensis*, and *T. dichotomus*, respectively. (A_3_–A_7_), (B_3_–B_7_), and (C_3_–C_7_) show cross-sectional morphologies of costal “C”, media posterior “MP”, the posterior end of “C”, wing membrane, and anal posterior “AP” of the three beetles, respectively.

The hind wings of the three beetles were folded in a V-shape when they were not in flight (Figure 3). For *P. brevitarsis*, *A. chinensis*, and *T. dichotomus*, body weight, body and extented hind wing lengths are increasing and are positively correlated (Table 1). In contrast, the flapping frequency is decreasing. Folding ratio, aspect ratio (i.e., the ratio between square of the wingspan and wing area), and the wing loading (i.e., the weight of the beetle divided by the wing area) of the three beetles were calculated. The folding ratio of *A. chinensis* was the smallest (0.34), and those of *P. brevitarsis* and *T. dichotomus* were similar (0.44 and 0.42, respectively). Aspect ratio and wing loading show an opposite trend.

Folding angle and folded states of the hind wings of three beetles are shown in Figure 3. The folding angle of the hind wings of *P. brevitarsis* is close to 120° (Figure 3A_2_), and the folding angle of the hind wings of *T. dichotomus* is close to 90° (Figure 3C_2_). The hind wings of *A. chinensis* overlapped when folded (Figure 3B_2_), and the folded length of the hind wings was smaller than the folded length of the other two species. Comparing the folding sites of the hind wings of the three beetles, the costal (C) and the media posterior (MP) of the hind wings of *P. brevitarsis* and *T. dichotomus* both were found to be folded, while the C of the hind wings of *A. chinensis* was found to be less folded, and the MP was not folded at the folding sites at all. This could be the reason for the folding ratio of the hind wings of *A. chinensis* being smaller.

Figure 3A_3_–A_7_, Figure 3B_3_–B_7_, and Figure 3C_3_–C_7_ show cross-sectional morphologies of different wing veins of the hind wings of three beetles at the same position obtained using SEM. The images show that the cross-sectional shapes are all nearly elliptical, while all were basically hollow, similar to blood vessels. This structure provides support for the beetles in spreading their hind wings or during flight [40]. Comparing the cross sections of different wing veins of the hind wings of the same beetles, the diameter of the C (Figure 3A_3_) was found to be larger than that of the MP (Figure 3A_4_) and the cubitus anterior (CuA) (Figure 3A_7_). Also, the diameter of the posterior end of the C (Figure 3A_5_) was smaller than that of the anterior end (Figure 3A_3_). Additionally, the wall thickness of the hind wing veins on the dorsal side was thicker than that on the ventral side. This was also found in the Asian ladybeetle (*Harmonia Axyridis*) [41]. Thicker wing veins are good for sustaining greater forces and preventing the hind wings from being damaged [42].

### Nanomechanical analysis of the hind wings

**Figure 4:**
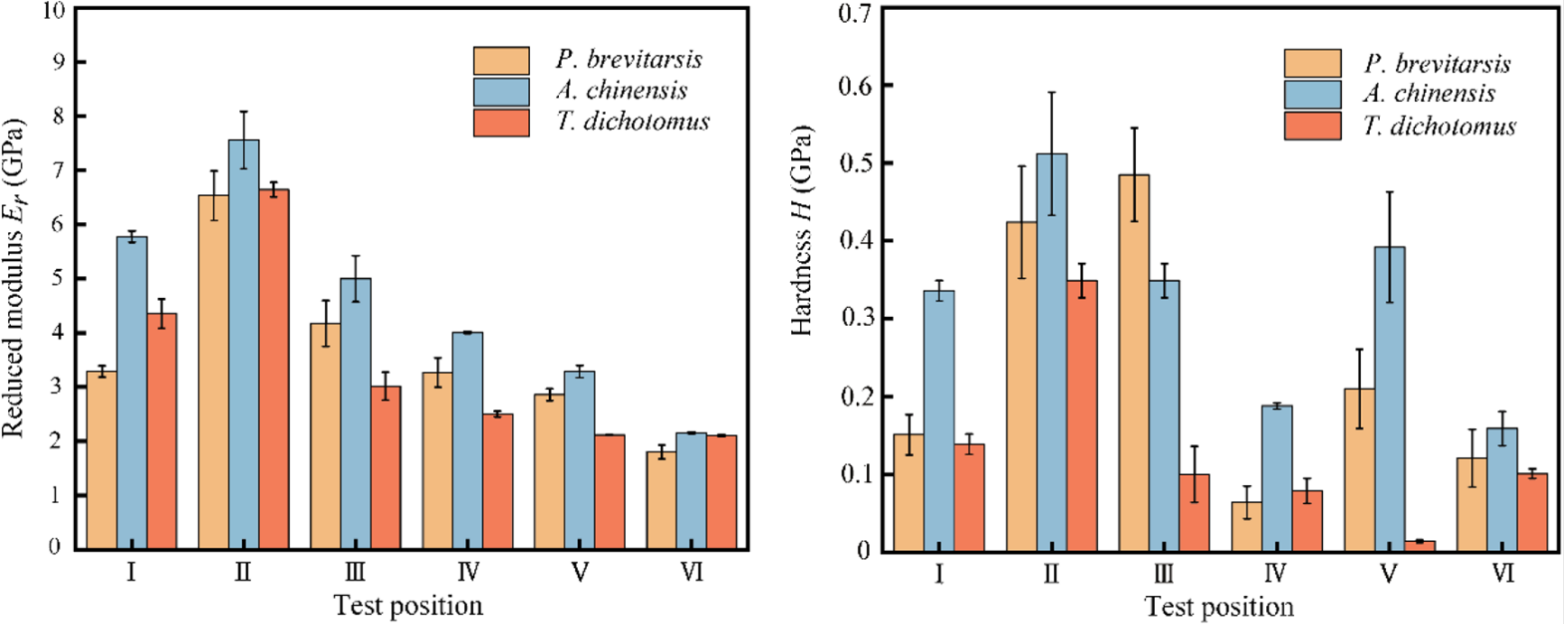
Nanomechanical test results of the anterior of the costal (I), the end of the costal (II), media posterior (III), cubitus anterior (IV), anal posterior (V), and wing membrane (VI).

Nanomechanical test results are shown in Figure 4. The nanomechanical properties of the hind wings of the three beetles change according to the same trend. The maximum values of the reduced Young’s modulus, *E*_r_, were all measured at test point II (the end of the costal) and were 6.530, 7.652, and 6.645 GPa for *P. brevitarsis*, *A. chinensis*, and *T. dichotomus*, respectively. The higher *E*_r_ helps the beetle to resist external forces and prevents wing damage [43]. The values of *E*_r_ and hardness *H* of cubitus anterior, anal posterior, and wing membrane were smaller than those of the costal and media posterior. It was also found that the *E*_r_ of costal of *P. brevitarsis* was smaller than those of *A. chinensis* and *T. dichotomus*, which possibly affected the deformation ability of the hind wing. Thus, the lift-to-drag ratio of *P. brevitarsis* was relatively large.

### Influence of wind speed, folding ratio, aspect ratio, and flapping frequency on the lift-to-drag ratio

**Figure 5:**
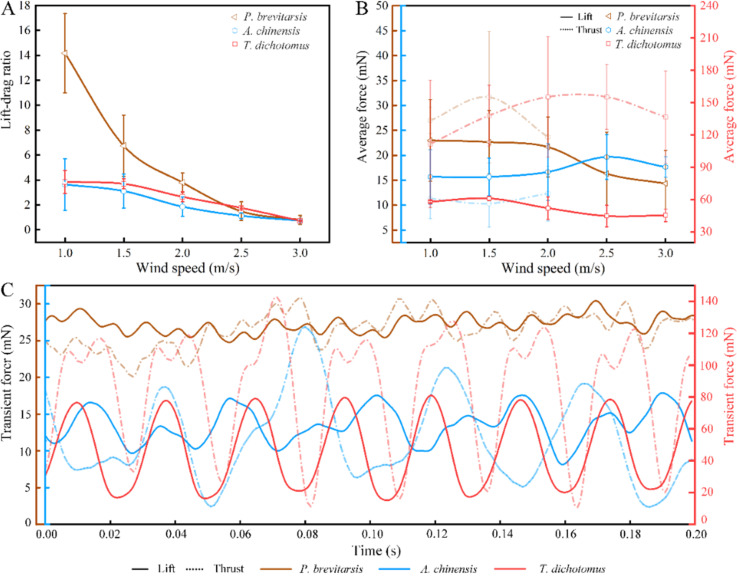
Aerodynamic characteristics of the three beetles. (A), (B), and (C) show the average lift-to-drag ratio, average aerodynamic force at different wind speeds, and the transient force curves of the three beetles (flapping time period of 0.2 s), respectively.

The wind tunnel test results of the three beetles at wind speeds of 1.0, 1.5, 2,0, 2.5, and 3.0 m/s are shown in Figure 5. With increasing wind speed, the lift-to-drag ratios of all three beetles show a decreasing trend (Figure 5A). When the wind speed increased, the drags of the beetles also increased, while the lift did not change significantly. At low wind speeds (<2.0 m/s), the drag on the beetle was small and the lift was relatively stable. Thus, the lift-to-drag ratio was higher than that at higher wind speeds (≥2.0 m/s). In addition, the lift-to-drag ratio of *P. brevitarsis* is obviously different from that of *A. chinensis* and *T. dichotomus*. It is significantly higher at low wind speeds and becomes gradually similar to the lift-to-drag ratio of the other two beetles at high wind speeds. For all three beetles, fluctuations of the lift-to-drag ratio were obvious at low wind speeds. At 1 m/s wind speed, the range of fluctuation of the lift-to-drag ratio was 10.5–16.0 for *P. brevitarsis*, 2.2–6.0 for *A. chinensis*, and 3.0–4.8 for *T. dichotomus.* At high wind speeds, the range of the lift-to-drag ratios gradually decreased and stabilized. For *P. brevitarsis*, although the downward trend of lift was not obvious (at low wind speed) (Figure 5B), the lift-to-drag ratio still rapidly descended. The reason for this is that, when the insect size decreased, the wing speed decreased (due to reduced wing length), while the wing drag increased (due to increased air viscosity) [44]. In addition, *P. brevitarsis* with a higher folding ratio was significantly affected by wind. Its peak lift force with increasing wind speeds appeared earlier than that of the other two beetles. This led to a larger decline of its lift-to-drag ratio compared to the other two beetles. So for *P. brevitarsis*, the effect of wind was greater than for the other beetles because of its small size. Considering only aspect ratio, wing area, or flapping frequency, the change trends of the lift-to-drag ratios of three beetles were not consistent. However, it was found that the lift-to-drag ratios of three beetles at low wind speeds varied directly with their folding ratio.

**Figure 6:**
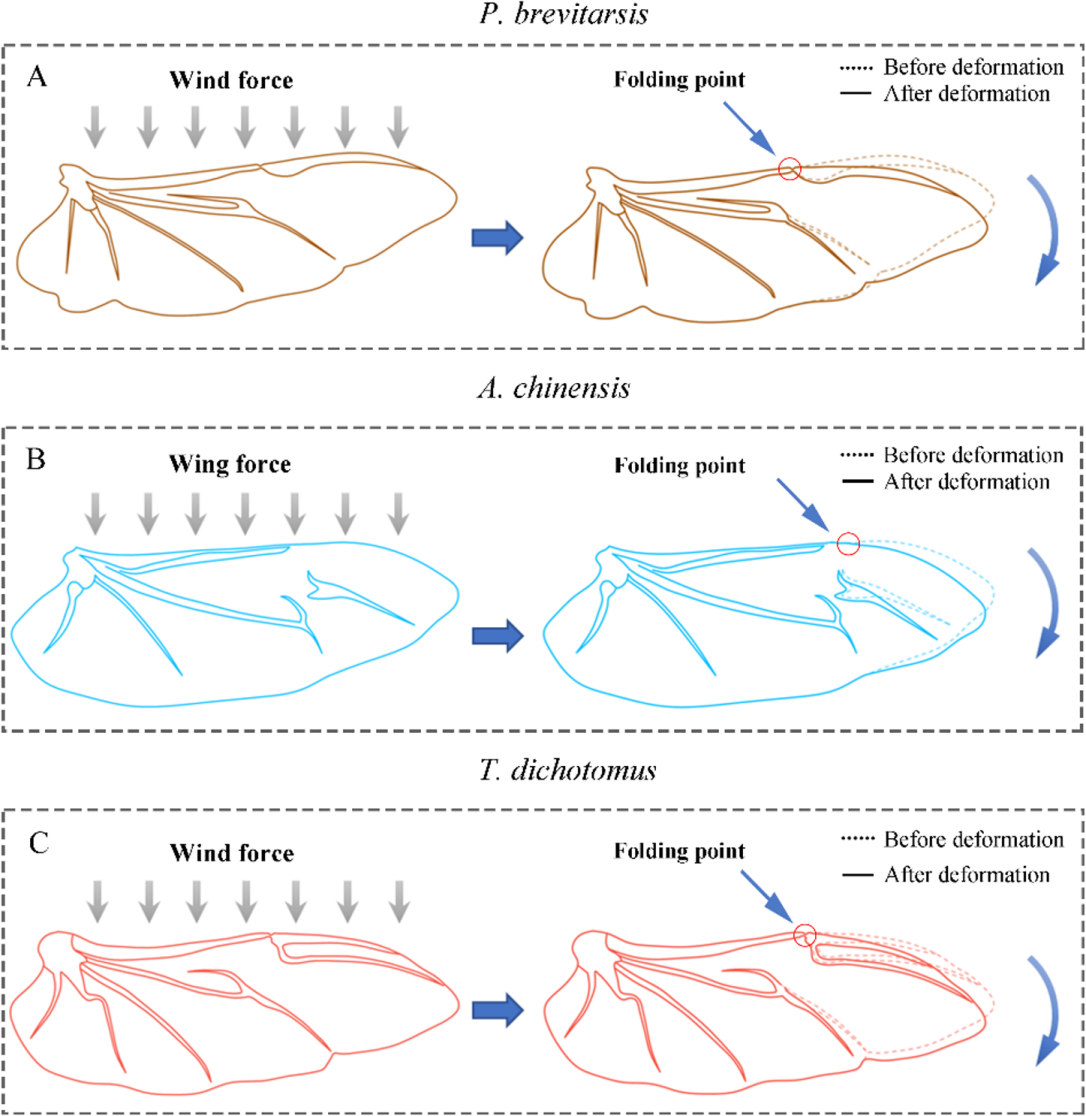
Schematic diagram of passive deformation of the hind wings of the three beetles.

The aerodynamic force at different wind speeds of the three beetles is shown in Figure 5B. It was found that the thrust of the beetles at different wind speeds was greater than their lift (except for *A. chinensis*). The lift of *T. dichotomus* was the biggest because its wing area was much larger than that of the other beetles. When the wind speed increased, the lift of three beetles wing in flapping motion first showed an increasing trend; but when the wind speed reached a certain value, the lift decreased. The drag increased with the wind speed. The order of the lift force peak values with increasing wind speeds was *P. brevitarsis*, *T. dichotomus*, and then *A. chinensis*. It was consistent with their folding ratio. A possible reason is that when the wind speed reaches a certain threshold, the hind wings would undergo passive deformation reducing the wing area, which results in decreased lift. A higher folding ratio of hind wings leads to easier deformation with increasing wind speed (Figure 6).

When the wind speed reached a certain value (Figure 5B) and the wings were flapping, lift and drag increased at the same time. At this time, the beetle could change its flight speed and obtain more lift or thrust to improve the flight efficiency and reduce the energy loss. When the wind speed reached a larger value, the lift decreased and the drag increased, and the aerodynamic performance of beetles was poor. At this time, continuing to maintain the original flight attitude and relying on the ability of the beetles to resist the wind causes insufficient lift to fly. Thus, at wind speeds greater than 2.0 m/s, there was no thrust for *P. brevitarsis* and *A. chinensis*. This means that, although the two beetles were flapping their hind wings, there was no forward movement. In nature, beetles could change lift, drag, and the moment of body rotation by changing the flight speed and the angle of attack to fly stably in different incoming flows and reduce energy consumption.

The transient force curves of the three beetles for a flapping time period of 0.2 s are shown in Figure 5C. The trends of lift and thrust curves of the three beetles show some convergence. As the lift of the beetle increases, the thrust also increases. In addition, the peak of lift and drag produced by *T. dichotomus* was the largest, up to 80 and 140 mN, respectively. Although *P. brevitarsis* is the smallest beetle, its peak lift and thrust were greater than that of *A. chinensis.* When the beetle is exposed to the wind force during flight, the hind wings undergo backward bending and passive deformation at the folding points, which directly decreases wing area (*S*_w_) and body frontal area (*S*_b_), which is helpful to reduce the profile drag and parasite drag (Figure 6).

The total drag on a beetle is equal to the sum of three aerodynamic components, as *D* = *D*_ind_ + *D*_pro_ + *D*_par_, where the components are induced drag *D*_ind_, profile drag *D*_pro_, and parasite drag *D*_par_. *D*_ind_ represents the cost of generating lift, *D*_pro_ is the drag of the wings and *D*_par_ is due to skin friction and the drag from the body form [45]. It was found that the wingspan *b* and *S*_w_ of the hind wings decrease simultaneously when passive deformation occurs. This changes the aspect ratio, *b*^2^/*S*_w_, and *D*_ind_ = 2*L*^2^/πρ*b*^2^*v*^2^). Thus, the drag reduction mainly comes from *D*_pro_ = 0.5ρ*v*^2^*S*_w_*C*_Dpro_ and *D*_par_ = 0.5ρ*v*^2^*S*_b_*C*_Dpar_, in which *C*_Dpro_ and *C*_Dpar_ are the dimensionless drag coefficient and the body drag coefficient, respectively. It might also be the reason for the high lift-to-drag ratio of *P. brevitarsis* at wind speeds lower than 2.0 m/s. The largest reduction of *S*_w_ and *S*_b_ led to the largest reduction in total drag compared to the other two beetles.

A multifactor analysis of variance was performed regarding the influence of folding ratio and wind speed on the lift-to-drag ratio. The results are shown in Table 3 (significance level is 0.05). The significance of the effect of the folding ratio and wind speed on the lift-to-drag ratio was less than 0.05, indicating that both folding ratio and wind speed would affect the lift-to-drag ratio of beetles. In this test, at low wind speeds (<2.0 m/s), the higher folding ratio was found to yield a better lift-to-drag ratio. With the increase of wind speed, the lift-to-drag ratio gradually decreased, and the effect of the folding ratio on the lift-to-drag ratio was no longer obvious.

**Table 3:** Tests of between-subjects effects.

Source	Type III sum of squares	df	Mean square	F	Sig.	Partial eta squared

corrected model	481.580^a^	14	34.399	20.324	0.000	0.905
intercept	513.760	1	513.760	303.543	0.000	0.910
folding ratio	94.102	2	47.051	27.799	0.000	0.650
wind speed	234.388	4	58.597	34.621	0.000	0.822
folding ratio × wind speed	153.090	8	19.136	11.306	0.000	0.751
error	50.766	30	1.693	–	–	–
total	1046.116	45	–	–	–	–
corrected total	532.356	44	–	–	–	–

^a^*R*^2^ = 0.905 (adjusted *R*^2^ = 0.860).

There is a correlation between aerodynamic forces and aspect ratio. Increasing the aspect ratio increases the average aerodynamic loads, and a smaller aspect ratio prevents the airflow from separating at the top and bottom [46]. Using butterfly wings as a prototype to design bionic wings with different aspect ratios and through wind tunnel tests and numerical simulations, it was found that a higher aspect ratio of bionic wings leads to a higher lift-to-drag ratio [19]. The three-dimensional effect of flow is weakened with increasing aspect ratio, which increases the aerodynamic coefficient [47]. Based on beetle hind wing models with different geometries, the average lift force was found to increase with increasing aspect ratio in a certain range [12]. Compared with the other two beetles, *P. brevitarsis* had a larger range of variation. This might be because the large aspect ratio of the wing yields a greater wing flexibility [48].

The flapping frequency of *P. brevitarsis*, *A. chinensis*, and *T. dichotomus* were 90, 45, and 43 Hz, respectively. It was found that *P. brevitarsis* had a higher flapping frequency and lift-to-drag ratio than the other beetles. The flapping frequencies of *A. chinensis* and *T. dichotomus* were similar. Especially, at low wind speeds, a higher flapping frequency yields a better lift-to-drag ratio. A bionic flexible FWMAV was tested in a wind tunnel to explore the effects of different flapping frequencies and aspect ratios on the aerodynamic performance. The flapping frequency played a crucial role in lift generation. Higher flapping frequencies yielded more lift. Also, a wing with a higher aspect ratio could significantly increase the lift [49]. For low flapping frequencies, the lift change is not obvious, but for high flapping frequencies, the lift increase is obvious [50]. The flapping frequency was found to affect inertial acceleration and aerodynamic pressure, thus affecting inertial force and aerodynamic force; with the increase of flapping frequency, their increment was relatively consistent [51]. For a four-wings FWMAV, the lift was increasing linearly with the flapping frequency [52]. Also, the increase in flapping frequency improves the thrust of the flapping [53].

Research on the living environments and living habits of the three beetles found that *P. brevitarsis* mostly live in farmland and loose soil, and the environmental conditions are relatively humid. Adults come out at night and have a strong flying ability. *P. brevitarsis* also has a strong migratory ability, moves quickly, and generally flies approximately 50 m [54–56]. *A. chinensis* lives mostly inside trunks of trees or in small closed forests, feeding on young leaves and bark [57]. *T. dichotomus* often lives in areas with well-developed forestry and thick trees und conditions native to a forest, feeding on the sap that flows from the surface of the bark. During the day, they mostly rest in shady and humid environments such as soil and move more in the evening hours [58–59]. Compared with the other two beetles, the living environment of *P. brevitarsis* is more open and offers a better flying environment. Long-term living habits improved its flying ability with better aerodynamic characteristics. Due to the narrow living environment, the flight performance of *A. chinensis* and *T. dichotomus* is not as good as that of *P. brevitarsis*. A study of the wing loading of the three beetles revealed that *P. brevitarsis* had the smallest wing loading of 19.85 g/dm^2^ matching its fast-moving nature. The wing loading of *A. chinensis* was 21.22 g/dm^2^, and the wing loading of *T. dichotomus* was 32.87 g/dm^2^. A lower wing loading leads to a better maneuverability [60]. Additionally, the wing loading affected the overload capacity of the aircraft, that is, the smaller the wing loading, the greater the overload of the aircraft [61]. Both *P. brevitarsis* and *A. chinensis* had smaller wing loadings, but *P. brevitarsis* had a higher lift-to-drag ratio, which might be due to higher folding ratio, aspect ratio, and flapping frequency. Although lift and drag of *P. brevitarsis* were more obviously affected by the wind speed, its excellent flight performance provides input for improving the maneuverability of MAVs.

## Conclusion

In this work, the effects of different hind wing parameters on the flight performance of beetles were studied through wind tunnel tests. Three beetle species with different living environments and different folding ratios were selected as the test objects in this study. The unfolded and folded morphology and the nanomechanical properties of the hind wings of the three beetles and the cross-sectional morphology of different wing veins were observed and tested with super depth-of-field microscopy, nanoindentation, and scanning electron microscopy. Thus, the folding ratios, reduced Young’s modulus, and hardness were determined. The flapping frequency was calculated by observing one flapping cycle with a high-speed camera. The effects of wind speed, folding ratio, aspect ratio, and flapping frequency on the lift-to-drag ratio of the beetles were examined in a low-speed straight-flow wind tunnel. The results showed that wind speed, folding ratio, aspect ratio, and flapping frequency had a combined effect on the flight performance of the beetles. Beetles with a more open living environment have a larger folding ratio, aspect ratio, flapping frequency, and wing loading, as well as better flight performance. The living environment of *P. brevitarsis* is relatively open, and its wing loading is also the smallest, reaching 19.85 g/dm^2^. This basic study will help to understand the effect of different beetle hind wing parameters on the flapping, which will be helpful for the design of biomimetic deployable FWMAVs.

## References

[1] Meresman Y, Husak J F, Ben-Shlomo R, Ribak G (2020). R Soc Open Sci.

[2] Kitagawa K, Sakakibara M, Yasuhara M (2009). J Visualization.

[3] Nguyen Q-V, Chan W L, Debiasi M (2015). Proc SPIE.

[4] Ho S, Nassef H, Pornsinsirirak N, Tai Y-C, Ho C-M (2003). Prog Aerosp Sci.

[5] Wu J, Sun M (2005). Acta Mech Sin.

[6] Nguyen Q-V, Chan W L, Debiasi M (2016). J Bionic Eng.

[7] Zheng L, Hedrick T, Mittal R (2013). Bioinspiration Biomimetics.

[8] Muhammad A, Nguyen Q V, Park H C, Hwang D Y, Byun D, Goo N S (2010). J Bionic Eng.

[9] Khan Z A, Agrawal S K (2011). AIAA J.

[10] Ha N S, Nguyen Q V, Goo N S, Park H C (2012). Exp Mech.

[11] Throneberry G, Hassanalian M, Abdelkefi A (2019). Drones.

[12] Liu C, Li P, Song F, Sun J (2021). Comput Biol Med.

[13] Sun J (2020). Science.

[14] Yang L J, Marimuthu S (2015). Acoustic comparison of PET and Latex wings for flapping micro-air-vehicles. IEEE 10th International Conference on Nano/Micro Engineered and Molecular Systems (NEMS).

[15] Phan H V, Park H C (2016). J Bionic Eng.

[16] Geisler T, Topczewska S (2015). Int J Appl Mech Eng.

[17] Geisler T (2016). Int J Appl Mech Eng.

[18] Wootton R J (1999). J Exp Biol.

[19] Rubio J E, Chakravarty U K (2019). Acta Mech.

[20] Du G, Sun M (2008). Appl Math Mech (Engl Ed).

[21] Bin Abas M F, Bin Mohd Rafie A S, Bin Yusoff H, Bin Ahmad K A (2016). Chin J Aeronaut.

[22] Lee B, Park H, Kim S-T (2015). J Mech Sci Technol.

[23] Geisler T (2012). Acta Mech Autom.

[24] Du J, Hao P (2018). Nanomaterials.

[25] Saito K, Yamamoto S, Maruyama M, Okabe Y (2014). Proc Natl Acad Sci U S A.

[26] Liu C, Li P, Song F, Stamhuis E J, Sun J (2022). Comput Biol Med.

[27] Dufour L, Owen K, Mintchev S, Floreano D (2016). A drone with insect-inspired folding wings. IEEE/RSJ International Conference on Intelligent Robots and Systems (IROS).

[28] Truong Q-T, Argyoganendro B W, Park H C (2014). J Bionic Eng.

[29] De Wagter C, Koopmans A, de Croon G, Remes B, Ruijsink R (2013). Autonomous Wind Tunnel Free-Flight of a Flapping Wing MAV. Advances in Aerospace Guidance, Navigation and Control.

[30] Lee B, Oh S, Choi H, Park H (2020). J Mech Sci Technol.

[31] Bomphrey R J, Nakata T, Henningsson P, Lin H-T (2016). Philos Trans R Soc, B.

[32] Meng X, Liu Y, Sun M (2017). J Bionic Eng.

[33] Zakaria M Y, Bayoumy A M, Elshabka A M (2009). Experimental Aerodynamic Characteristics of Flapping Membrane Wings. 13th International Conference on aerospace science.

[34] Liu L, Zhang X J, He Z X J (2012). Theor Appl Inf Technol.

[35] Chen P, Joshi S, Swartz S M, Breuer K S (2014). Bat-Inspired Flapping Flight. 22nd AIAA/ASME/AHS Adaptive Structures Conference.

[36] Nian P, Song B, Xuan J, Yang W, Dong Y (2019). IEEE Access.

[37] Truong T V, Le T Q, Byun D, Park H C, Kim M (2012). J Bionic Eng.

[38] Phan H V, Truong Q T, Park H C (2019). Bioinspiration Biomimetics.

[39] Johansson L C, Engel S, Baird E, Dacke M, Muijres F T, Hedenström A (2012). J R Soc, Interface.

[40] Sun J, Liu C, Bhushan B, Wu W, Tong J (2018). Beilstein J Nanotechnol.

[41] Sun J Y, Liu C, Du R J, Zhang Z J (2017). Microstructural characteristics and nanomechanical properties of hind wings of the Asian ladybeetle, harmonia axyridis. 017 7th International Conference on Manipulation, Manufacturing and Measurement on the Nanoscale (3M-NANO).

[42] Sun J, Wu W, Ling M, Bhushan B, Tong J (2016). Beilstein J Nanotechnol.

[43] Sun J, Liu C, Bhushan B (2019). J Mech Behav Biomed Mater.

[44] Lyu Y Z, Sun M (2021). J Insect Physiol.

[45] Hedenstrom A, Liechti F (2001). J Exp Biol.

[46] Shademan M, Naghib-Lahouti A (2020). Adv Aerodyn.

[47] Luo G, Sun M (2005). Acta Mech Sin.

[48] Ryu Y, Chang J W, Chung J (2019). Aerosp Sci Technol.

[49] Deng S, Percin M, van Oudheusden B, Remes B, Bijl H (2014). Int J Micro Air Veh.

[50] Jongerius S R, Lentink D (2010). Exp Mech.

[51] Yang W, Song B, Song W, Wang L (2012). Chin Sci Bull.

[52] Cheng C, Wu J, Zhang Y, Li H, Zhou C (2020). Adv Aerodyn.

[53] Tan X, Zhang W, Ke X, Zou C, Liu W, Cui F, Wu X S, Li H Y (2012). Development of Flapping-wing Micro Aerial vehicle in Asia. Proceedings of the 10th World Congress on Intelligent Control and Automation (WCICA 2012).

[54] Lowrey S, De Silva L, Hodgkinson I, Leader J (2007). J Opt Soc Am A.

[55] Miao J, Wu Y-Q, Li K-B, Jiang Y-L, Gong Z-j, Duan Y, Li T (2015). J Insect Behav.

[56] Hegedüs R, Szél G, Horváth G (2006). Vision Res.

[57] Hérard F, Maspero M (2019). J Pest Sci.

[58] Kojima W, Ishikawa Y, Takanashi T (2014). Naturwissenschaften.

[59] Hongo Y (2006). J Ethol.

[60] Terblanche J S, Anderson B (2010). Naturwissenschaften.

[61] Almbro M, Kullberg C (2012). J Insect Behav.

